# A call for consistency and integration in global surface water estimates

**DOI:** 10.1088/1748-9326/ad1722

**Published:** 2024-01-16

**Authors:** Adnan Rajib, Arushi Khare, Heather E Golden, Bikas C Gupta, Qiusheng Wu, Charles R Lane, Jay R Christensen, Qianjin Zheng, Travis A Dahl, Jodi L Ryder, Brian C McFall

**Affiliations:** 1 Hydrology & Hydroinformatics Innovation Lab, Department of Civil Engineering, University of Texas, Arlington, TX, United States of America; 2 U.S. Environmental Protection Agency, Office of Research and Development, Cincinnati, OH, United States of America; 3 U.S. Environmental Protection Agency, Office of Research and Development, Athens, GA, United States of America; 4 Department of Geography, University of Tennessee, Knoxville, TN, United States of America; 5 U.S. Army Corps of Engineers, Engineer Research and Development Center (ERDC) Coastal and Hydraulics Laboratory, Vicksburg, MS, United States of America; 6 U.S. Army Corps of Engineers, Engineer Research and Development Center (ERDC) Environmental Laboratory, Vicksburg, MS, United States of America

**Keywords:** global surface water, Earth observations, open science, lakes and wetlands, water security, surface water mapping

Global shifts in climatic conditions and anthropogenic disturbances are dramatically modifying the Earth’s ecosystem. Impacts from these changes are evident in the fluctuation, degradation and destruction of surface waters, and lack of sustainability in water consumption (Lane *et al* 2023). Ensuring future water security within this context will largely depend on how accurately we can map Global Surface Water Extents (GSWEs) (Cooley *et al* 2021). The availability of accurate GSWE data can improve understanding of hydrological, biological, and biogeochemical processes in watersheds, reduce uncertainties in flood and drought predictions, and advance conservation and restoration policy decisions.

Mapping GSWE is not necessarily a new science, but it is rapidly evolving. Nearly two decades ago Lehner and Döll (2004) developed the first comprehensive open dataset—the Global Lakes and Wetlands Database—mapping perennial water bodies with extents ⩾0.1 km^2^. The authors merged myriad older generation maps, such as the World Register of Dams and Wetland Maps of the World Conservation Monitoring Centre, to develop the dataset. While highly advanced for the time, these maps captured nearly 3 million km^2^ of water bodies worldwide, covering only 40% (by area) of the current global estimates (Pekel *et al* 2016).

Since the development of the Global Lakes and Wetlands Database (Lehner and Döll 2004), there has been a Big Data revolution, expanding our competency in Earth observations and geospatial informatics. Concomitantly, mapping GSWE with greater spatial detail has become increasingly feasible. Earth observation-based GSWE datasets have emerged as a result, with millions of previously unaccounted water bodies now mapped. More recently, various global land cover datasets have shifted GSWE mapping capabilities to an unprecedented 10 m spatial resolution (Brown *et al* 2022). Some of these new generation GSWE data incorporate value-added attributes, mapping temporal dynamics such as inundation frequency and seasonality of surface waters (Pickens *et al* 2020).

With the abundance of new global datasets, we ask: how different are the currently available GSWE datasets and what directions do we—as a water research and management community—need to go to improve their consistency and integration? The answers to these questions will reveal critical insights to ensure optimal GSWE selection for targeted water resources research, management, and policy goals, now and into the future. On the surface, many GSWE data look similar: they emerged at nearly the same time, used identical source data, and even adopted similar names, e.g. Global Surface Water (GSW, Pekel *et al* 2016), Global Inland Surface Water (GIW, Feng *et al* 2016), Global 3-s Water Body Map (G3WBM, Yamazaki *et al* 2015), and Global Water Body (GLOWABO, Verpoorter *et al* 2014). However, inconsistencies exist across GSWE data-sets, and as a result, their respective estimates of water area extents may vary. Yet this critical issue has remained largely unaddressed in the scientific literature. Therefore, understanding the relative inconsistencies among GSWE datasets and how this influences their applications for future science, management, and policy questions demands immediate attention, particularly as these data continue to evolve.

Here, we suggest that currently available GSWE datasets—despite their inconsistencies—are a foundation from which to improve the development, ease-of-use, and applicability of GSWEs into the future. We posit, however, that more strategic, transparent, and accessible approaches are necessary to select the best GSWE dataset or to integrate multiple suitable GSWE datasets for specific research, management, and policy questions and decisions. We therefore propose an integrated open science framework as a hub for GSWE data and a means to facilitate GSWE data interoperability with multiple cross-disciplinary modeling, analysis, synthesis, and data-visualization tools. Thus, bringing all GSWE datasets under one global framework will lead to a wholistic, convergent understanding of the climate-water-human nexus.

## 1. Global surface water datasets are inconsistent

**Figure 1.**
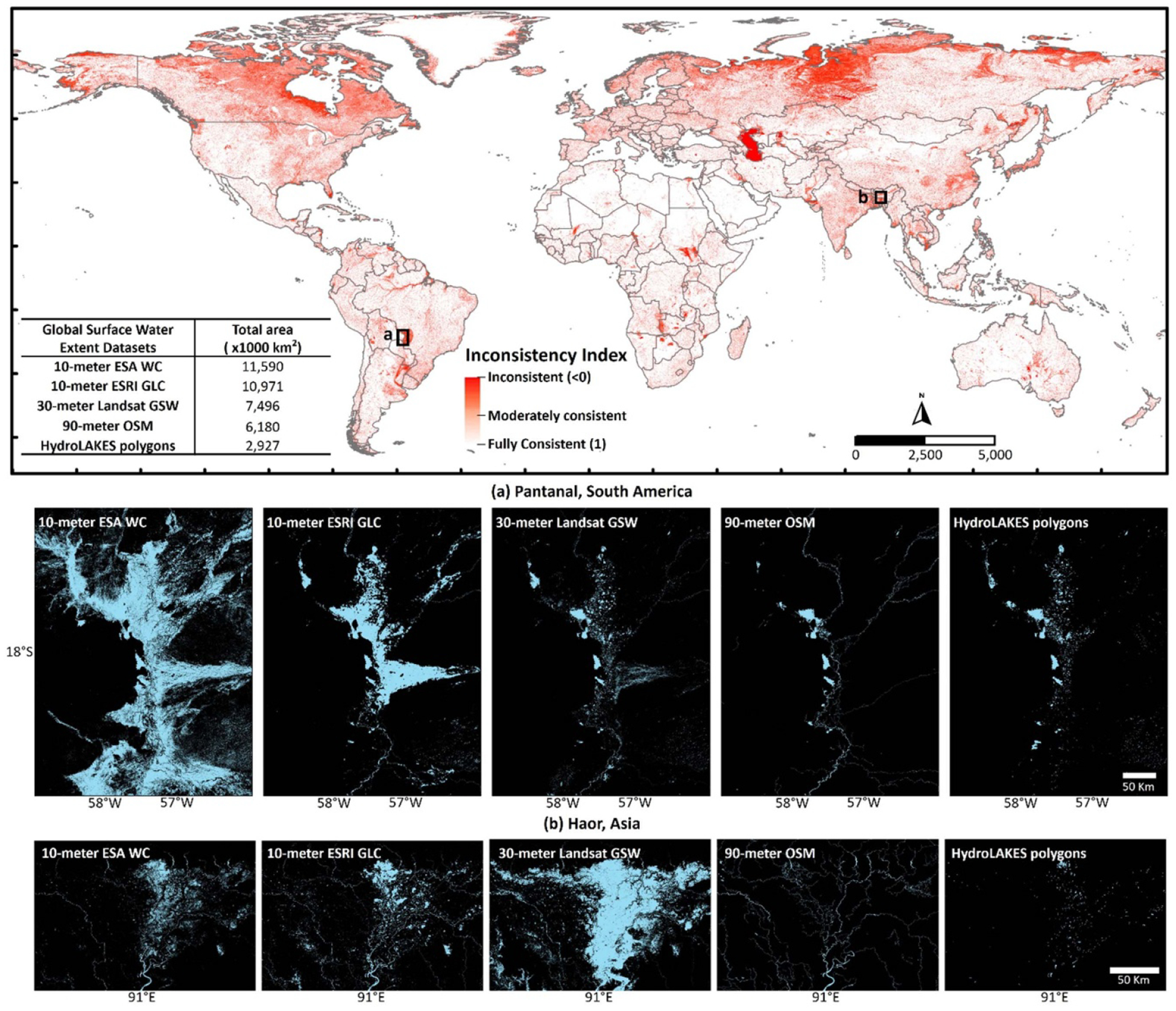
Currently available Global Surface Water Extent (GSWE) estimates are highly inconsistent due to differences in their data origin, data format, and definitions of surface waters. The global map presents inconsistency across five contemporary GSWE datasets in the form of an index (Falotico and Quatto 2015), calculated for year 2020 at every 1 km of the world. (a) and (b) GSWE inconsistencies in some of the world’s most hydro-ecologically significant regions show how higher spatial resolution of the data does not correspond to better mapped surface water extents. The data shown here can be visualized and compared through a newly developed open science framework Global Surface Water Information System (GSWIS) (Rajib *et al* 2023): https://gswis.gishub.org. Here, ESA WC = European Space Agency WorldCover (Zanaga *et al* 2021), ESRI GLC = Environmental Systems Research Institute Global Land Cover (Karra *et al* 2021), Landsat GSW = Landsat Global Surface Water (Pekel *et al* 2016), OSM = OpenStreetMap (Yamazaki *et al* 2019), HydroLAKES polygons (Messager *et al* 2016).

An underlying theme across GSWE datasets is their relative inconsistencies, which stem from three root causes: (1) data origins, (2) data formats, and (3) data-specific nomenclature and definitions of ‘surface water’ (table 1). As we demonstrate in figure 1, these differences impact our estimates of global surface water resources.

Currently, GSWE datasets originate from one of three broad source categories: (1) merged hydrography, (2) Landsat satellite imagery, or (3) Sentinel satellite imagery. All three vary in their spatial-temporal resolutions. Merged hydrography synthesizing various static maps and surveys of global hydrography has remained the most traditional way of producing GSWE datasets (Messager *et al* 2016). Landsat-based GSWE data first emerged as static estimates of permanent water extent and have evolved into estimates of long-term dynamics at global scale (Verpoorter *et al* 2014, Yamazaki *et al* 2015, Feng *et al* 2016, Pekel *et al* 2016, Pickens *et al* 2020, JRC 2023). Landsat-based GSWE have further evolved into frequent, temporally continuous estimates at regional scales (Jones 2019). GSWE data from Sentinel satellites are relatively recent developments, providing continuous estimates and at higher spatial resolutions than Landsat (Karra *et al* 2021, Zanaga *et al* 2021). Other emerging approaches show promise for surface water mapping, yet they add more variability into how a GSWE dataset is chosen for a specific field of application. For example, large basin hydrologic models of floods, droughts, and water quality are increasingly using GSWE derived from topography data (Rajib *et al* 2020). Very high-resolution data from commercial satellites can detect up to 90% additional surface waters missed by Landsat (Vanderhoof *et al* 2019)—a valuable resource limited to local applications. Many of these GSWE datasets have visually comparable spatial distributions of surface waters, yet these visual similarities do not equate to commensurate total area. For example, during the same time-period of 2020, European Space Agency (ESA) WorldCover (Zanaga *et al* 2021) detects 11.5 million km^2^ of total surface water extent across the globe whereas OpenStreetMap (OSM; Yamazaki *et al* 2019) detects 6.1 million km^2^ (figure 1). Structural differences in respective data origins contribute to such large variations.

**Figure 2.**
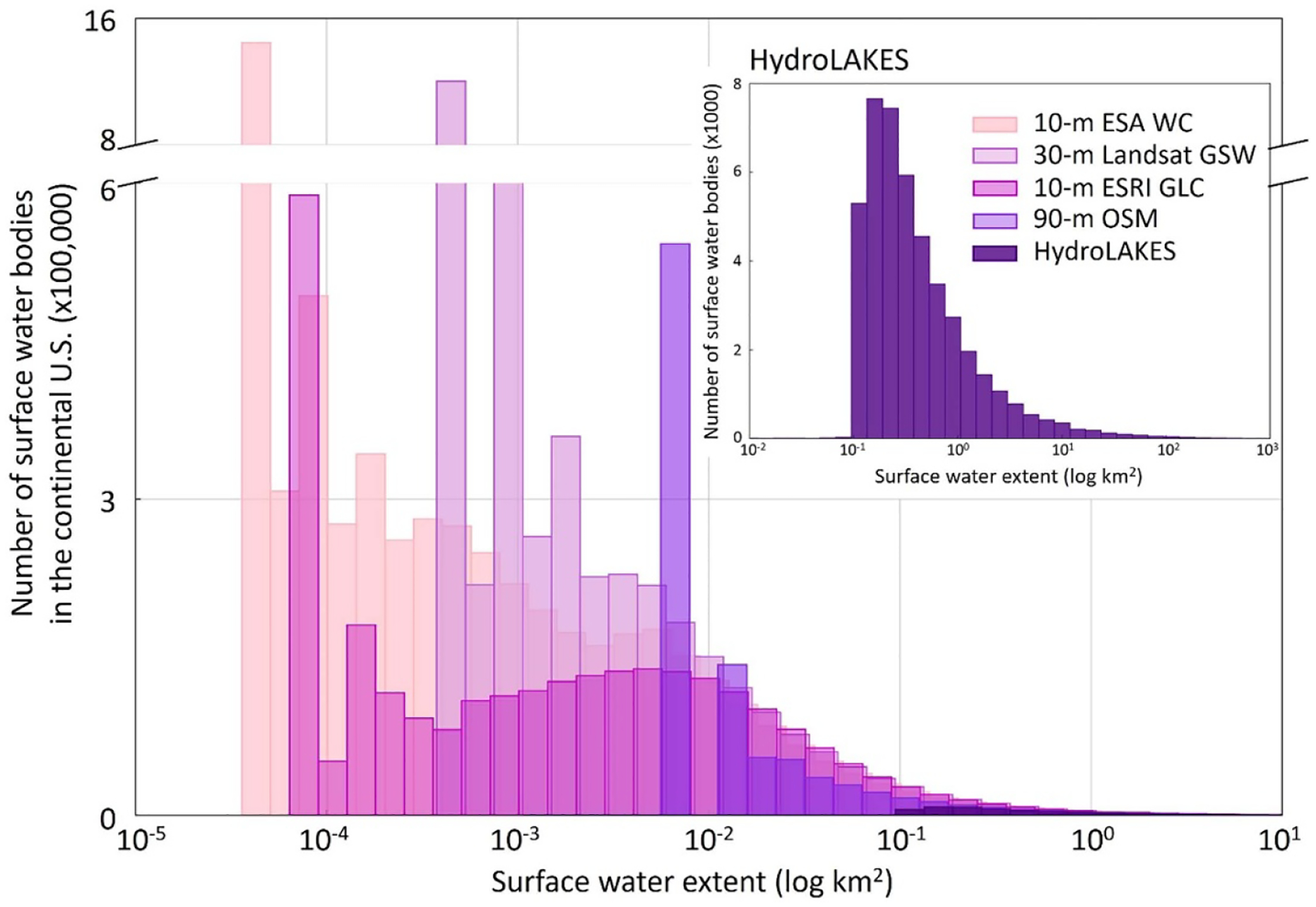
Inconsistency across five contemporary Global Surface Water Extent (GSWE) datasets within the continental U.S. demonstrated as frequency distributions of different water body sizes. All data correspond to year 2020, except HydroLAKES which is not associated with any specific year.

Existing GSWE datasets have inconsistent data formats, which result in different size distributions (figure 2). For example, Lake Mead, a nationally significant hydropower reservoir in the U.S., is 350 km^2^ in the ESA WorldCover gridded data (Zanaga *et al* 2021). The same water body is 40% larger in the HydroLAKES polygon data (Messager *et al* 2016), thus contributing to the variations in size distributions across datasets regionally and globally. While the non-overlapping time-period of mapping is a reason for these variations (WorldCover in figures 1–2 represents year 2020 while HydroLAKES does not identify a specific time period), these variations are further pronounced when format transformations are performed for a one-on-comparison between two datasets.

Each GSWE dataset defines ‘surface waters’ differently (table 1). Unlike the inconsistencies in data origin and data format, variations in data-specific nomenclature and definitions of surface waters are not readily obvious. Some GSWE datasets (1) present wetlands and flooded vegetation as separate classes from permanent waters (Karra *et al* 2021, Zanaga *et al* 2021), (2) distinguish rivers, lakes, and small streams but do not include wetlands (Yamazaki *et al* 2019), or (3) simply detect any surface inundation (Verpoorter *et al* 2014, Yamazaki *et al* 2015, Feng *et al* 2016, Pekel *et al* 2016, Pickens *et al* 2020, JRC 2023). When specific descriptions of surface waters are overlooked, critical errors in assessments of global water availability can emerge. For example, despite originating from the same Sentinel data source as ESA WorldCover (Zanaga *et al* 2021), Environmental Systems Research Institute Global Land Cover (ESRI GLC, Karra *et al* 2021) yields 5.5% underestimation of total surface water extent globally for the year 2020 (figure 1). The methodological differences in defining surface waters between the two datasets attribute to this underestimation. In another example, compared to its 10 m Sentinel counterparts (Karra *et al* 2021, Zanaga *et al* 2021), 30 m Landsat-based GSW dataset of 2020 (Pekel *et al* 2016) depicts nearly 40 times more inundation for the smaller water bodies in the continental U.S. (size range 0.01–0.001 km^2^) (figure 2) despite underestimating the total surface water extent globally (figure 1). This is because the GSW and other Landsat-based data define surface waters more liberally than Sentinel-based data which include a distinct classification of water or water-covered surfaces.

We therefore hypothesize that variations in data origins, formats, nomenclature and definitions—which produce inconsistencies across GSWE datasets—negate simplistic calls for increased GSWE spatial resolutions. Moving from 90 m to 10 m data will not necessarily correlate with accurate GSWE estimates. Further, there is no ‘true’ baseline GSWE dataset from which to compare others. Specifically, datasets with coarser spatial resolutions may provide information more aligned with that required to answer a specific management or research question. For example, deriving continuous estimates for research questions related to global or regional surface water extents may be too computationally intensive if 10 m data are used. Using coarser estimates may be more efficient in this situation. Similarly, using GSWE datasets representing specific water body types, like large lakes and reservoirs (HydroLAKES), does not necessarily equate to an underrepresentation of water bodies if the research or management target is, in fact, focused on large water body systems.

To improve appropriate selection and use of GSWE data, all characteristics of the GSWE data (e.g. data origin, spatial and temporal resolutions, data format, and nomenclature and definitions; table 1) need to be considered. Specific research or management questions should drive the selection of a dataset or integration of multiple datasets.

## 2. The future entails more divergence and complexity in global surface water data

The current GSWE mapping efforts are pushing the limits of Earth observations to attain unprecedented temporal frequency and spatial resolution. Meanwhile, we are entering a new era of Earth observations where continuously monitoring surface water levels would be feasible (Chen *et al* 2022). Preliminary proofs of concept demonstrate how combining these water level observations with coincident area extents can estimate changes in global water availability with greater precision (Cooley *et al* 2021). It seems plausible to even envision a future with 3D surface water datasets by leveraging the next-generation topographic data products including Lidar point clouds, digital surface, and terrain models (USGS 3DEP 2022). While these advancements are promising, they are also setting the stage for a future with more divergence and complexity in surface water data. However, without ensuring consistency and integration in today’s GSWE datasets, a stride towards future developments will not be meaningful. Housing the GSWE data in one framework will simplify this process.

## 3. The solution: an integrated global surface water framework

Today’s GSWE mapping effort do not follow the findable, accessible, interoperable, and reusable (Wilkinson *et al* 2016) principle which weakens their application-readiness. Specifically, these datasets are findable and accessible, but lack in interoperability and reusability potentials because a unified data standard or protocol of GSWE mapping does not exist. There is also a dearth of metadata and supporting documentation that could explain the inconsistencies across these datasets. Combined, these issues make it challenging to harmonize and integrate GSWE datasets into environmental management, policy, and research applications. The good news is some of these limitations are already being addressed by the scientific community such as the LAGOS-LOCUS platform developed for the U.S. lake ecology community (Cheruvelil *et al* 2021). Yet these recent developments remain at regional scales, are geared towards very specific community needs, and may not be reproducible globally.

We propose to fill this gap by integrating all GSWE datasets under one global framework. We demonstrate this through a newly developed open science framework—the Global Surface Water Information System (GSWIS) (Rajib *et al* 2023) which enables users to instantaneously and interactively inspect the inconsistencies across different GSWE datasets by doing a one-on-one and side-by-side comparison using cloud computing resources. We envision that, this prototype framework, when fully developed, will let users select any region of interest to analyze surface water extents and inundation dynamics using graphs and statistical results.

The scientific community must produce surface water data in ways that are interoperable and reusable by scientists, decision-makers, and the public. Bringing all GSWE datasets under one open science framework will lead us to that direction and pave the way to new tools and software solutions (Song *et al* 2023) that will facilitate rapid linking of GSWE data with other hydroclimatologic and socio-economic data repositories and models (Meyer *et al* 2020). This will truly enable convergent research, management, and policy decisions in climate-water-human nexus, thus increasing our information capability to successfully address water security challenges of the 21st century.

## Figures and Tables

**Table 1.** Contemporary Global Surface Water Extent (GSWE) datasets.

Name and origin^a^	Spatial-temporal^b^ resolution	Nomenclature and definitions of surface water
Merged hydrography		
HydroLAKES (Messager *et al* 2016)	⩾0.1 km^2^, static	Global lakes
Landsat-based		
GLOWABO (Verpoorter *et al* 2014)	15 m, static (1999–2002)	Global lakes >0.002 km^2^
GIW (Feng *et al* 2016)	30 m, static (1999–2003)	Inland fresh and saline waterbodies >0.005 km^2^
G3WBM (Yamazaki *et al* 2015)	90 m, dynamic (1987–2012)	Permanent waterbodies and temporal water-covered areas
GSW (Pekel *et al* 2016, JRC 2023)	30 m, dynamic (1984–2021)	Maximum extent, seasonality, occurrence, recurrence, transitions, yearly water history
GSWD (Pickens *et al* 2020)	30 m, dynamic (1999–2021)	Permanent and stable seasonal water, loss and gain, dry and wet periods
Sentinel-based		
ESA WorldCover (Zanaga *et al* 2021)	10 m, continuous (2020, 2021)	Permanent water bodies, herbaceous wetlands, and mangroves
Dynamic World (Brown *et al* 2022)	10 m, continuous (2015–present)	Surface waterbodies as water and flooded vegetation
ESRI Global Land Cover (Karra *et al* 2021)	10 m, continuous (2017–2022)	Surface waterbodies as water and flooded vegetation
Others		
OSM (Yamazaki *et al* 2019)	90 m, static (2020)	Large lakes and rivers, major rivers, canals, and minor streams

a GLOWABO = Global Water Body, GIW = Global Inland Surface Water, G3WBM = Global 3-s Water Body Map, GSW = Global Surface Water, GSWD = Global Surface Water Dynamics, ESA = European Space Agency, ESRI = Environmental Systems Research Institute, OSM = OpenStreetMap.

b Static = a single estimate of long-term average condition; Dynamic = statistics of long-term temporal dynamics; Continuous = continuous estimates at frequent intervals. All are gridded datasets, except HydroLAKES (which is available as boundary polygons).

## Data Availability

The data that support the findings of this study are openly available at the following URL/DOI: https://gswis.gishub.org.
